# Cinemeducation: a descriptive mixed-methods analysis of perspectives in a medical humanities course

**DOI:** 10.1080/10872981.2025.2579077

**Published:** 2025-11-18

**Authors:** Moritz Trieb, Lisa M. Pfadenhauer, Céline Kohll, Martin R. Fischer, Matthias Siebeck, Mike Rueb

**Affiliations:** a Institute of Medical Education, LMU University Hospital, LMU Munich, Munich, Germany; b Institute of Medical Information Processing, Biometry and Epidemiology (IBE), Faculty of Medicine, LMU Munich, Munich, Germany; c Pettenkofer School of Public Health, Munich, Germany; d Department of Obstetrics and Gynaecology, TUM University Hospital rechts der Isar, Munich, Germany; e Department of Psychiatry and Neurosciences, Charité Campus Mitte, Charité – Universitätsmedizin Berlin, Berlin, Germany; f Department of Psychiatry and Psychotherapy, Charité at St. Hedwig Hospital, Charité – Universitätsmedizin Berlin, Berlin, Germany

**Keywords:** Cinemeducation, medical humanities, arts and humanities, medical education, interprofessional, film, cinema, lived experience

## Abstract

**Introduction:**

Co–organized by students, the cinemeducation course ‘M23 Cinema’ (M23C) at Ludwig–Maximilians–Universität Munich combines film screenings with audience discussions featuring guests to promote perspective-taking and reflective thinking on health. This study assessed the M23C’s inclusivity regarding film representation, guest diversity, and organizing committee composition, considering gender, profession, institution, academic background, and geographic origin.

**Methods:**

This mixed–methods study pursued a descriptive qualitative and quantitative analysis of all M23C events from 2006 to 2024. We used a database of internal and publicly available records on film characteristics, thematic content, and demographics of guests and organizers.

**Results:**

Among 103 M23C events, 66 (64.1%) were feature films and 36 (35.0%) documentaries, addressing 80 topics, with abortion, assisted dying, and organ transplantation being the most frequent. Films originated from 29 countries, with 99 (97.1%) (co–)produced in the Global North. Of 231 guests, 119 (51.5%) were physicians from 33 specialties, 45 (19.5%) were people with lived experience or relatives, and 42 (18.2%) were other health professionals. Most guests (55%) were male. Among 39 organizing committee members, 37 (94.9%) were medical students, and 26 (66.7%) were female.

**Discussion:**

This study demonstrates the potential of cinemeducation to address diversity and representation in medical education. We identified gaps, including Eurocentrism and gender imbalances, highlighting the need for a more inclusive approach. Nine practical implications developed to improve inclusivity, including incorporating people with lived experiences, balancing gender representation, integrating global health perspectives, diverse student groups, and fostering interdisciplinary collaboration through film festivals and professional networks.

## Introduction

Clinicians in Germany and globally are becoming increasingly aware of diverse patient populations [1]. Diversity spans age and gender, as well as cultural and ethnic backgrounds, sexual orientations, and other characteristics. Minority groups that were previously overlooked are increasingly able to voice their specific healthcare needs, which often vary across different communities [2]. Medical professionals must consider diverse needs, values, and expectations to provide adequate healthcare [3]. Therefore, it is necessary to build competencies and skills in healthcare professionals that enhance patient–centered care, where treatments and interactions align with patients’ unique experiences and values [3,4]. A key competency in delivering such tailored care is the ability to engage in *perspective–taking*, the ability to adopt the psychological view of others [5,6]. By gaining a nuanced understanding of patients’ and colleagues’ backgrounds, values, and experiences, the quality of care can be improved [7]. Conversely, unconscious biases and stereotypes can negatively impact decision–making and exacerbate health disparities [8]. Previous studies suggest that developing perspective–taking skills promotes empathy and drives patient–centered care that directly benefits patient outcomes [8–10].

Perspective–taking should encompass diverse viewpoints, including those of patients, interprofessional healthcare teams (*e.g.,* nursing, psychology, social work), and professionals from fields beyond healthcare. Interprofessional collaboration strengthens access to comprehensive, patient–centered care [11,12]. At the same time, transdisciplinary approaches—drawing from law, political or social science, or the arts—can inspire innovative solutions to complex health problems [13,14]. Showing multiple perspectives can facilitate the acquisition of cultural competencies by encouraging students to reflect on different values, beliefs, and healthcare experiences [15]. Additionally, incorporating insights from people with lived experience (individuals who have personally experienced illness, disability, or the healthcare system) ensures that healthcare strategies are grounded in real–world realities [16].

Biases, such as Eurocentrism in medicine, further highlight the need for inclusivity. Examples include the unequal distribution of COVID−19 vaccines and cultural insensitivity towards migrant patients [17–19]. Such biases perpetuate stereotypes, discriminatory behavior, and inequities [20]. Similarly, the underrepresentation of women, particularly in leadership roles, underscores systemic barriers for women and other marginalized groups in healthcare [21]. In addition to these indirect effects, the history of Western medicine also includes instances of direct harm, such as unethical medical experimentation, forced sterilizations, and the exploitation of colonized populations [22–24]. Acknowledging this problematic legacy by integrating diverse perspectives is essential to achieving equitable patient–centered care [25].

Reflective learning is critical for developing perspective–taking skills. *Cinemeducation*—a method that combines cinema, medicine, and education—presents a valuable tool [26]. Introduced in the early 1980s, cinemeducation uses feature films, documentaries, and series to prompt discussions on complex medical and social topics [27], such as addiction [28] and end–of–life care [29]. Research indicates that this approach is often more engaging than traditional educational methods—such as lectures, textbook–based instructions, or case–based discussions—as its emotional narratives effectively address sensitive topics [30,31]. There are indications that emotional learning enhances long–term recall of knowledge [32].

The M23 Cinema (M23C), established at Ludwig–Maximilians–Universität (LMU) Munich, Germany, applies this cinemeducation approach [32,33]. The M23C course features film screenings followed by moderated audience discussions involving medical professionals, professionals from non–health backgrounds, and those directly (*e.g.,* patients) or indirectly (*e.g.,* relatives) affected by health issues. The courses occur three to four times per semester and are held in a lecture hall equipped for audiovisual presentations. Each event lasts approximately 2.5 hours, including the film screening and a 45-minute discussion. While medical students make up the core audience, sessions are open to students from other disciplines and faculties. Embracing the idea of co–design, the course is organized by a student–led committee selecting films and guests. Previous evaluations of the M23C have shown that it fosters reflective learning, perspective–taking, and emotional engagement [33], contributing to a deeper understanding of biopsychosocial aspects of health and illness [32].

In this study, we analyzed the M23C curriculum from 2006 to 2024 to evaluate the degree of inclusive representation. Specifically, we assessed the characteristics of the (i) films, (ii) the invited guests, and the (iii) organizing committee, focusing on gender, profession, institution, academic background, and geographic representation.

## Methods

We conducted a descriptive mixed methods study based on a document analysis to examine and analyze our documented chronology of all M23C events. As it serves multiple purposes, including offering context, raising queries, complementing other research data, monitoring changes over time, and validating other sources, this method was the most suitable approach to analyzing the available data [34,35]. Document analysis, as described by Dalgish, entails four steps, namely (1) ready your materials, (2) extraction of data, (3) analysis of data, and (4) distill your findings [35].

### Setting

This study was conducted at the medical faculty at LMU Munich in Germany. The cinemeducation course M23C was delivered between 12/01/2006 and 23/01/2024.

### Data collection

To *ready* our materials as described in step one [35], we created a database by collecting relevant documents to reconstruct the 103 cinemeducation events. These documents entailed official invitation e–mails and posters, online announcements published on the website [www.m23kino.de], and information shared across two social media channels (*i.e.,* Instagram ‘@m23_kino’ and Facebook ‘M23Kino’). The information included in these documents was extracted and compiled in a matrix (see Table 1, Supplementary File 7). The extracted data for each M23C event comprised year, term (summer/winter), date of M23C event, format (in–person/online via Zoom), film title in original language, English film title, director, director’s gender, film release year, production country (a film could be co–produced by multiple countries), film category (*i.e.*, documentary, feature film, novel), film duration, topic (*e.g.,* abortion, euthanasia), and the discussion guests with selected demographic data (*i.e.,* profession, institution, academic background, gender). The extracted data (number, profession, gender) of the former and current student committee members were obtained from the website.

After extraction, we applied a set of predefined categories based on our research questions and the event structure (deductive coding). During the coding process, we further refined and expanded these categories through inductive analysis, allowing additional sub–categories to emerge directly from the data [35]. To ensure consistency and rigor, MT and MR independently coded a subset of the data and resolved discrepancies through iterative discussions. The coding framework was continuously refined, and final categories were agreed upon by all authors by consensus.

Potential mismatches in the compiled data were resolved through conversations with former and current organizing committee members and the authors (*e.g.,* missing data on experts’ gender). MR translated the extracted data from German to English, which MT checked (see Supplementary File 1).

### Data analysis

The data collected in the table was subsequently descriptively analyzed and visualized in R [36]. We analyzed the professional backgrounds of the guests, categorizing them as follows: 1) Physicians (including medical students), 2) other health professionals, 3) non–health professionals, 4) people with lived experience or relatives, and 5) film team. The gender demographics of M23C guests, directors, and the organizing committee were classified as male, female, or diverse (including trans, intersex, and unknown). For the subgroup analyses regarding gender, we calculated the confidence intervals (CI) of the gender variables. We assessed the invited physicians’ specialties and the guests’ academic degrees.

Furthermore, we analyzed the origin of the films by considering the country or countries of (co–)production. The country of production often signifies where the film was financed, providing insights into the perspectives presented [37]. These countries were categorized into six world regions, and the Global North or the Global South. Additionally, we examined the film categories, including feature films, documentaries, and other formats.

### Ethical approval

Ethical approval for the study was not required due to a retrospective analysis of already open–access data.

### Results

A total of 103 (*n* = 103) M23C events were included in the analysis (see Table 1). Of these, 98 (95.1%) were delivered in person in a lecture hall, and 5 (4.9%) were delivered online due to the COVID−19 pandemic.

**Table 1.** Overview of all M23C events.

English film title	Director	Year	Duration (min)	Topic	Guest I: Profession	Guest II: Profession	Guest III: Profession	Guest IV: Profession	Guest V: Profession
The Sea Inside	Alejandro Amenábar	2004	124	Euthanasia	Law	Paediatrics	Gynaecology and obstetrics	Cinema operator	
Time to Leave	François Ozon	2005	81	Cancer	Paediatrics				
The Sea Inside	Alejandro Amenábar	2004	125	Euthanasia	Palliative medicine				
Coma	Michael Crichton	1978	113	Organ transplantation	Transplant surgery				
The Diving Bell and the Butterfly	Julian Schnabel	2007	112	Locked-in syndrome	Nursing	Neurology			
Knockin’ on Heaven’s Door	Thomas Jahn	1997	89	Death	Haematology and oncology				
Away from Her	Sarah Polley	2006	110	Dementia	Psychiatry				
The Stranger in Me	Emily Atef	2008	99	Postpartum depression	Psychiatry	Gynaecology and obstetrics			
Sicko	Michael Moore	2007	123	US healthcare system	Occupational medicine				
4 Months, 3 Weeks and 2 Days	Cristian Mungiu	2007	113	Abortion	Social pedagogy, social work				
Peas at 5:30	Lars Büchel	2004	112	Blindness	Person with lived experience				
XXY	Lucía Puenzo	2007	87	Intersexuality	Paediatrics				
The Constant Gardener	Fernando Meirelles	2005	129	Drug testing in Africa	Tropical medicine				
My Sister’s Keeper	Nick Cassavetes	2009	109	Stem cell transplantation	Paediatrics				
Adam's Apples	Anders Thomas Jensen	2005	94	Sex offenders	Forensic psychiatry				
Living in Emergency: Stories of Doctors Without Borders	Mark N. Hopkins	2008	93	Doctors without borders	Family medicine				
The Heart of Jenin	Marcus Vetter, Leon Geller	2008	89	Organ transplantation	Transplant surgery				
Vincent Wants to Sea	Ralf Huettner	2010	96	Tourette syndrome	Child and adolescent psychiatry				
Million Dollar Baby	Clint Eastwood	2004	127	Spiritual Care	Psychosomatics				
Requiem for a Dream	Darren Arnofsky	2000	97	Drug addiction	Psychiatry				
Thank You for Smoking	Jason Reitman	2005	92	Smoking	Pharmacology and toxicology				
Dr. Alemán	Tom Schreiber	2008	106	Gang violence	Neurology				
Blindness	José Saramago	1995		Blindness	Audiobook speaker				
Eternal Sunshine of the Spotless Mind	Michel Gondry	2004	104	Memories	Psychology				
Biutiful	Alejandro González Iñárritu	2010	148	Medical care without health insurance	German studies	Social pedagogy, social work			
Me Too	Álvaro Pastor Gaspar, Antonio Naharro	2009	103	Down syndrome	Paediatrics				
Milk	Gus Van Sant	2008	128	Homosexuality	Medical didactics				
Beyond Silence	Caroline Link	1996	112	Deafness	Pedagogy for the deaf and hearing impaired	Pedagogy for the deaf and hearing impaired	Relative		
The Intouchables	Olivier Nakache, Éric Toledano	2011	112	Quadriplegia	Neurology				
Super Size Me	Morgan Spurlock	2004	100	Fast food	Paediatrics				
Amour	Michael Haneke	2012	127	Death	Palliative medicine				
50/50	Jonathan Levine	2011	100	Brain tumour	Neurology	Person with lived experience			
Juno	Jason Reitman	2007	96	Teenage pregnancy	Social pedagogy, social work	Gynaecology and obstetrics			
Same Same But Different	Detlev Buck	2009	106	HIV, AIDS	Psychology	Person with lived experience			
I Am a Woman Now	Michiel van Erp	2011	80	Transidentity	Urology	Person with lived experience	Person with lived experience	Person with lived experience	
Rust and Bone	Jacques Audiard	2012	122	Amputation	Vascular surgery	Orthopaedic technology	Physiotherapy	Person with lived experience	
Outing	Sebastian Meise, Thomas Reider	2012	76	Paedophilia	Psychology				
At The Doctor's Side – Stéphane & Franziska	Sylviane Gindrat	2013	52	Family medicine	Family medicine	Family medicine	Family medicine	Family medicine	
In a Better World	Susanne Bier	2010	113	Development aid	Child and adolescent psychiatry				
Same But Different	David Barnes	2013	59	Dealing with illnesses, disabilities and limitations from a child's perspective	Paediatrics	Nursing			
Fire In The Blood	Dylan Mohan Gray	2012	87	Access to medication	Medical student	Nursing	Pedagogy		
The Fault In Our Stars	Josh Boone	2014	129	Psycho-oncology for palliative tumour patients in children and adolescents	Palliative medicine	Psychology	Relative	Relative	
Living in Emergency: Stories of Doctors Without Borders	Mark N. Hopkins	2008	93	Doctors without borders	Family medicine				
Monsoon Baby	Andreas Kleinert	2014	89	Surrogacy	Gynaecology and obstetrics	Film production			
One Flew Over The Cuckoo’s Nest	Miloš Forman	1975	123	Stigmatisation of psychiatry	Child and adolescent psychiatry	Psychiatry			
Talk to Her	Pedro Almodóvar	2002	112	Coma	Anaesthesia	Nursing			
Tour de Force	Christian Zübert	2014	95	Amyotrophic lateral sclerosis	Palliative medicine	Neurology	Social pedagogy, social work		
Born This Way	Shaun Kadlec, Deb Tullmann, Jamie Wolf	2013	82	LGBT in Africa	Medical didactics				
Simply being normal	Felix Julian Koch	2015	30	Sailing rebels	Haematology and oncology	Person with lived experience	Person with lived experience	Person with lived experience	
Still Alice	Richard Glatzer, Wash Westmoreland	2014	101	Dementia	Biology	Neurology	Person with lived experience	Relative	
Extraordinary Measures	Tom Vaughan	2010	106	Pompe disease	Neurology	Pharmaceutical industry	Person with lived experience	Relative	
Requiem For A Dream	Darren Aronofsky	2000	97	Drug addiction	Social pedagogy, social work				
Precious	Lee Daniels	2009	110	Sexual abuse	Forensic psychiatry	Psychology	Psychology		
Tales From the Organ Trade	Ric Esther Bienstock	2013	82	Organ transplantation	Surgery	Person with lived experience			
The doctor who thinks around the corner	Jule Sommer, Udo Kilimann	2015	43	Rare diseases	Cardiology				
Ebola: The Doctor’s Story	Steven Grandison	2016	41	Ebola	Tropical medicine	Nursing	Virology		
Soul Birds	Thomas Riedelsheimer	2009	90	Stem cell transplantation	Social pedagogy, social work	Haematology and oncology	Person with lived experience	Person with lived experience	
The Danish Girl	Tom Hooper	2015	120	Transidentity	Urology	Child and adolescent psychiatry	Person with lived experience	Person with lived experience	
24 Weeks	Anne Zohra Berrached	2016	103	Abortion	Medical ethics	Gynaecology and obstetrics	Relative	Person with lived experience	
Kinsey	Bill Condon	2004	118	Sexuality and self-determination	Psychology	Gynaecology and obstetrics	Neurology	Urology	
Obesity: The Post Mortem	Melanie Archer	2016	55	Obesity	Epidemiology	Visceral surgery	Endocrinology	Dietician	
The Checklist Effect	Lauren Anders Brown	2016	45	Checklists	Anaesthesia	Nursing			
Notes on Blindness	Pete Middleton, James Spinney	2016	90	Blindness	Ophthalmology	Person with lived experience	Person with lived experience		
On Call	Alice Diop	2016	96	Migration	Surgery	Philosophy			
Dallas Buyers Club	Jean-Marc Vallée	2013	117	HIV, AIDS	Virology	Pharmacology and toxicology			
Picco	Philip Koch	2010	105	Prison	Forensic psychiatry	Civil servant	Psychology	Psychology	
The Unknown Girl	Jean-Pierre Dardenne, Luc Dardenne	2016	106	Professionalism	Medical ethics	Psychosomatics	Medical didactics		
Multiple Fates – About the Struggle with One's Own Body	Jann Kessler	2016	84	Multiple sclerosis	Film direction	Neurology	Administrative sciences	Person with lived experience	Person with lived experience
The Kindness Of Strangers	Maro Chermayeff	1998	100	Organ transplantation	Psychiatry	Visceral surgery	Person with lived experience		
Sugar Coated	Michèle Hozer	2015	91	Sugar	Gastroenterology	Paediatrics	Diabetology		
The Third Option	Thomas Fürhapter	2017	78	Prenatal diagnostics	Medical ethics				
The Immortal Life Of Henrietta Lacks	George Costello Wolfe	2017	93	HeLa cells	Biology	Pharmacology and toxicology			
Code Black	Ryan McGarry	2013	81	Emergency room	Emergency medicine				
trustWHO	Lilian Franck	2018	85	World Health Organization	Political science	Public health	Medical didactics		
4 Kings	Theresa von Eltz	2015	98	Adolescent psychiatry	unknown				
FEMMEfille	Kiki Allgeier	2014	87	Anorexia nervosa	Psychology	Child and adolescent psychiatry			
#Female Pleasure	Barbara Miller	2018	97	Sexuality and self-determination	Philosophy	Psychology	Gender studies		
Like Father, Like Son	Hirokazu Koreeda	2013	120	Adoption	Psychology	Person with lived experience	Person with lived experience		
The market-orientated patient	Leslie Franke, Herdolor Lorenz	2018	82	Healthcare system	Health economics	Medical ethics	Nursing		
Vessel	Diana Whitten	2014	90	Abortion	Medical doctor	Social pedagogy, social work			
XXY	Lucía Puenzo	2007	87	Intersexuality	Paediatrics	Person with lived experience	Person with lived experience		
Shame	Steve McQueen	2011	100	Sex addiction	Neurology	Alternative practitioner	Psychology		
Trashed	Candida Brady	2012	98	Environmental pollution	Anaesthesia	Medical student	Medical student		
We tick differently – living with Tourette's	37 Grad, ZDF	2020	30	Tourette syndrome	Psychiatry	Person with lived experience			
I die the way I want	37 Grad, ZDF	2020	30	Euthanasia	Palliative medicine	Medical ethics	Law		
In love, engaged, beaten up – violence against women	Daniela Agostini	2019	45	Domestic violence	Social pedagogy, social work	Forensic medicine			
System Crasher	Nora Fingscheidt	2019	120	System crasher	Child and adolescent psychiatry	Psychology			
Desert Flower	Sherry Hormann	2009	121	Female genital mutilation	Person with lived experience	unknown	Gynaecology and obstetrics		
Lost in Face	Valentin Riedl	2021	81	Prosopagnosia	Film direction	Person with lived experience			
The Father	Florian Zeller	2020	98	Dementia	Biology	Social pedagogy, social work	Relative		
Homeopathy Unrefuted?	Erik Lemke	2021	86	Homeopathy	Medical didactics	Pharmacology and toxicology	Paediatrics		
The Miseducation of Cameron Post	Desiree Akhavan	2018	90	Conversion therapy	Law	Person with lived experience			
Another Round	Thomas Vinterberg	2021	116	Alcohol	Person with lived experience	Pharmacology and toxicology			
The Fever	Katharina Weingartner	2019	99	Malaria	Tropical medicine	Tropical medicine			
Everything Went Fine	François Ozon	2021	113	Euthanasia	Psychiatry	Palliative medicine	Law	Medical ethics	
The White Sound	Hans Weingartner	2001	106	Schizophrenia	Psychiatry	Social pedagogy, social work	Person with lived experience		
Beautiful Boy	Felix Van Groeningen	2018	121	Drug addiction	Psychiatry	Social pedagogy, social work	Person with lived experience		
Fog in August	Kai Wessel	2016	126	Medicine under National Socialism	Psychiatry	Psychiatry	Relative		
The Intouchables	Olivier Nakache, Éric Toledano	2011	112	Humour in medicine	Palliative medicine	Acting			
Everything Everywhere All At Once	Daniel Kwan, Daniel Scheinert	2022	139	Depression	Psychiatry	Psychology			
Happening	Audrey Diwan	2022	100	Abortion	Medical doctor	Medical ethics	Social pedagogy, social work		
The Peanut Butter Falcon	Tyler Nilson, Michael Schwartz	2019	98	Disability and inclusion	Pedagogy	Paediatrics	Relative	Social pedagogy, social work	
The Whale	Darren Aronofsky	2022	117	Obesity	Cardiology	Person with lived experience	Person with lived experience		

#### Representation in films

##### Thematic focus of films

In 103 M23C events, 80 different topics were addressed. The topics most frequently addressed in the M23C (*n* = 4 each) were abortion, assisted dying, and organ transplantation. Dementia, blindness, and drug addiction were addressed three times (more detailed analysis in Supplementary File 2). On one occasion (0.9%), the film was substituted with a ‘reading in the dark’ experience reflecting on blindness; everything else was unchanged.

##### Category of film

Of the 103 M23C events, 66 (64.1%) were feature films, and 36 (35.0%) were documentaries (see Table 1).

##### Production countries and regions

Of the 102 films shown at the M23C, the production companies originated from 29 countries (see Supplementary File 3A). 34 (33.3%) from the USA, 31 (30.4%) from Germany, and 13 (12.7%) from France. In some cases (*n* = 19), companies from several countries were involved in the film production (cp. Supplementary File 4). 36 (35.3%) were (co–)produced by German–speaking countries (*i.e.*, Germany, Austria, Switzerland). Most films were (co–)produced by European (*n* = 66, 64,7%) or North American (*n* = 38, 37,2%) production companies (see Supplementary File 3B). Thus, 99 (97.1%) were (co–)produced in the Global North.

##### Gender representation of directors

**Figure 1.**
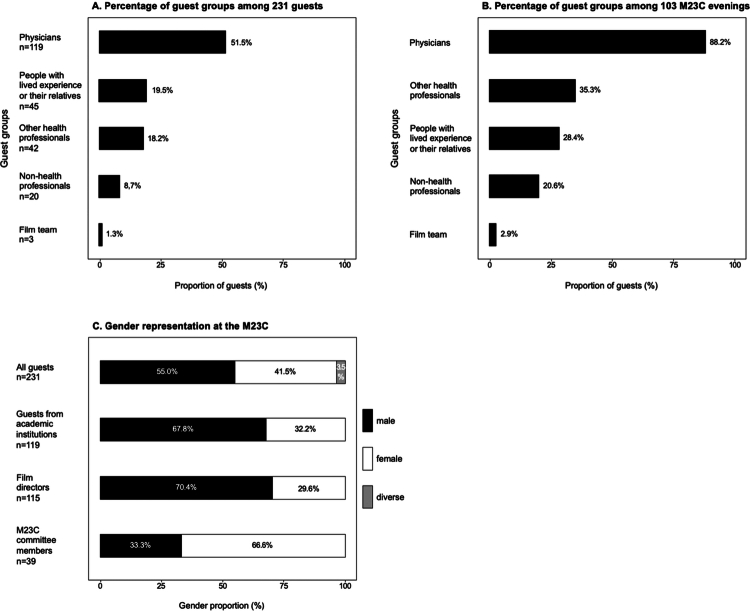
**Composition of guest groups and gender representation in the M23C curriculum.** Panel A depicts the distribution of guest groups among all guests (*n* = 231) in the M23C Cinema (M23C) program. Physicians constitute the largest group (*n* = 119, 51.5%), followed by people with lived experience or their relatives (*n* = 45, 19.5%), other health professionals (*n* = 42, 18.2%), non–health professionals (*n* = 20, 8.7%), and people from the film team (*n* = 3, 1.3%). For two guests, the profession was unknown. Panel B demonstrates the proportion of guest groups across all M23C events (*n* = 103), with physicians being guests in 90 (88.2%), other health professionals in 36 (35.3%), people with lived experience or their relatives in 29 (28.4%), non–health professionals in 21 (20.6%), and film team in 3 (2.9%). Panel C illustrates gender representation among M23C participants and directors of the films shown, indicating a predominance of male representation among all guests (55.0% of *n* = 231), especially among academic institution affiliates (67.8% of *n* = 119) and film directors (70.4% of *n* = 115). In contrast, the M23C student committee is predominantly female (67.6% of *n* = 39).

115 film directors were involved in the 102 films (some in pairs or threes). Of these, 81 (70.4%) were male and 34 (29.6%) were female (see Figure 1C).

#### Representation of guests

In total, 231 guests were invited to 103 M23C events.

##### Professional background of guests

Regarding profession, most guests were trained as physicians (*n* = 119; see Figure 1A and B), followed by other health professionals (*n* = 42). More details in Supplementary File 5.

**Figure 2.**
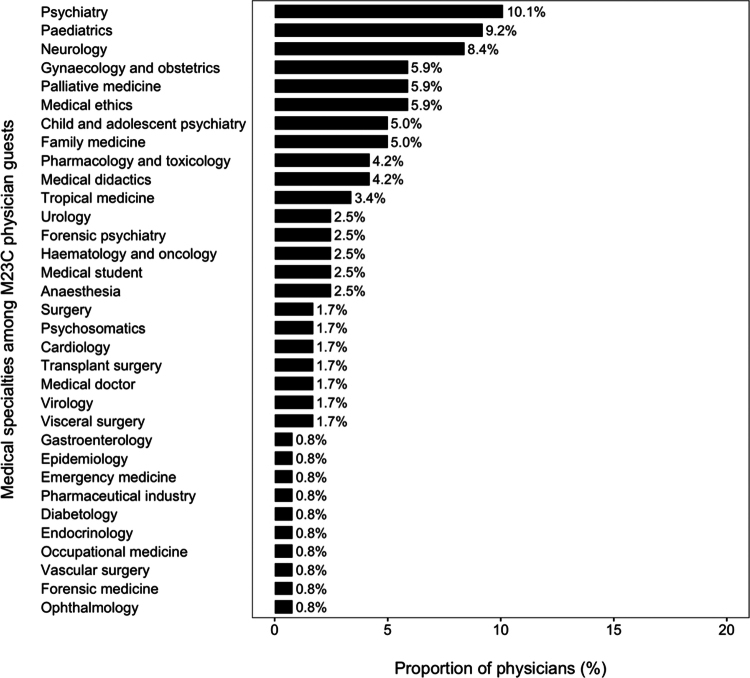
Medical specialty of M23C physician guests.

The 119 physicians who attended the M23C as guests represented 33 medical specialties (see Figure 2).

##### Academic background of guests

In total, 130 (56.3%) of the 231 guests had an academic degree: 58 (25.1%) had a professorship, 55 (23.8%) a doctorate, and 17 (7.4%) a habilitation (academic qualification with permission to teach and supervise university students).

##### Gender of guests

Of 231 guests, 127 (55.0%, CI: 48.3%–61.5%) were male, 96 (41.5%, CI: 35.2%–48.2%) female, and 8 (3.5%, CI: 1.6%–7.0%) categorized as diverse (see Figure 2C). For guests (*n* = 118) representing academic institutions, 80 (67.8%, CI: 58.5%–75.9%) were male, and 38 (32.2%, CI: 24.1%–41.5%) female, see Table S4. Of 119 invited medical doctors, 81 (68.1%, CI: 58.8%–76.1%) were male, and 38 (31.9%, CI: 23.9%–41.2%) female (see Supplementary File 6).

#### Representation in the organizing committee

Among the 39 organizing committee members, 37 (94.9%) were medical students. One was also an intensive care nurse, another studied physics, and another pursued political science. One member was a dental student, and one was a physician. 26 (66.7%) were female, and 13 (33.3%) were male (see Figure 2C).

### Discussion

This study analyzed all M23C events concerning representation in three domains: the films screened, the guests participating in audience discussions, and the composition of the organizing committee. The analysis highlighted the suitability of the cinemeducation methodology employed in the M23C for addressing aspects of diversity and representation. Diverse health–related topics were explored primarily through feature films and documentaries, with most films produced in Europe or North America. This pattern also holds for other cinemeducation curricula [38,39]. Most guests were identified as male and physicians who held advanced degrees. In contrast, most organizing committee members were medical students and female.

The M23C, with nearly two decades of experience and over 100 film events, offers a broader range of medical topics than previously published cinemeducation courses [26,27,38–41]. Our M23C program demonstrates that cinemeducation can be applied to nearly all medical topics, given the availability of suitable films. The committee included diverse media formats, prioritizing feature films to attract a larger audience, but has also intentionally included more documentaries since 2013. This demonstrates that feature films, documentaries, short films, or series can be used for cinemeducation if they address medical narratives and spark a discussion among students. We also propose video essays as a promising addition to this methodology [42].

The gender distribution among the guests exhibits an imbalance, characterized by an overrepresentation of guests identified as male relative to female or diverse. The overrepresentation of male physicians from academic institutions reflects Germany’s gendered medical hierarchies [43,44] and aligns with intersectionality theory’s critique of how power consolidates around dominant identities [45].

The M23C has shown more than twice as many films by male directors as female directors. This can be explained, on the one hand, by an already–known gender imbalance within the film industry [46] and, on the other hand, by a male perspective in programming. Given that most team members are female, a gender bias seems unlikely. Instead, it points to a broader structural gender issue within the film industry [47].

We had no data on the proportion of minorities and vulnerable groups on the guests or organizing committee (*e.g.,* queer, (post-)migrant, first–generation medical students, and people of color). We can imagine that these diverse perspectives in the organizing committee can lead to more varied and pluralistic programming, ultimately benefiting the students in their training as transformative change agents [48].

Most guests were physicians, likely due to the organizing committee’s consistent effort to invite a physician to every M23C event, and most of the committee being medical students. Overrepresentation of physicians risks promoting a predominantly biomedical view of health and illness [49]. In contrast, the lower participation of people with lived experience, relatives, and other healthcare professionals highlights the need for the M23C to incorporate more interprofessional and patient perspectives in future guest selections. Interprofessional and patient perspectives enhance education by fostering collaboration and a more comprehensive, patient–centered approach to healthcare. Since not all M23C events focus on illness, including a person with lived experience in every session may not always be appropriate. Additionally, finding suitable affected persons for specific conditions proved to be a challenge.

The higher proportion of guests with academic degrees can be attributed to the university setting, which underscores the clinical and scientific rigor of the information presented to the students.

The predominance of guests with psychiatric or psychological backgrounds, along with the frequent focus on psychiatric topics in M23C, aligns with international trends [50]. This suggests that psychiatric themes are especially well–suited for illustrating the biopsychosocial approach to medicine through cinemeducation [38]. While psychiatric topics were well–covered, the films in the M23C shown so far rarely explored how race or migration status intersect with mental health.

The comparatively low percentage of non–health professionals as guests suggests that transdisciplinary perspectives are considered in the curation of M23C events but remain underrepresented. This was also because it took more effort to persuade other professional groups to participate in a medical course.

When looking at the countries from which the films were (co–)produced, it became clear that most of the films—and presumably also the perspectives and narratives shown—are Eurocentric or by the Global North and high–income countries. Even if a film was co–produced by the Global South, this does not mean that the perspectives of the Global South are sufficiently represented in the film. This may reflect film consumption habits in Germany, where audiences predominantly engage with film productions from the Global North, especially North America.

Given the M23C’s multi–perspective approach and the increasing importance of global health issues such as pandemics, planetary health, and migration health, physicians in the Global North must adopt a more comprehensive understanding of health challenges. To achieve this, it is essential that films from low– and middle–income countries, particularly those from the Global South, be included in cinemeducation curricula moving forward. It is vital to assess whether merely including films from the Global South is adequate or if appropriate representation should be ensured within the organizing committee and among guests.

A representative and diverse cinemeducation course enhances the medical curriculum by facilitating reflective thinking on personal sociocultural backgrounds and the structural privileges inherent in the medical profession [51]. Integrating diverse perspectives within cinemeducation broadens students’ understanding of health and fosters the development of a humanistic, patient–centered approach [40]. Furthermore, it promotes cultural competence, empathy, and the ability to engage with complex societal dimensions of healthcare, ultimately preparing students to address health disparities and serve diverse patient populations more effectively [52].

Given their inherently transnational, transmedia, and interdisciplinary nature, film festivals provide an ideal setting for students and developers of cinemeducation curricula to discover diverse and representative films [53].

Our cinemeducation framework, published in 2024 [33], emphasizes film–based learning in medical education through reflective thinking, perspective–taking, and emotional narratives. Compared to the recently published prism model [54], which provides a refined theoretical approach to integrating arts and humanities across medical education. While developed independently, both frameworks share overlapping dimensions: for example, ‘gaining knowledge’ aligns with ‘mastering skills,’ ‘perspective–taking’ appears in both, ‘attitudes’ and ‘opinions’ correspond to ‘personal insights,’ and ‘reflective thinking’ overlaps with ‘social advocacy.’ Together, these two frameworks can guide the representative curation of film and guest selection for cinemeducation.

#### Implications for practice for cinemeducation curricula

After analyzing and interpreting the qualitative and quantitative data, we discussed the results. We identified nine implications for practice for the film and guest selection and organizing committee of the M23C and other cinemeducation and arts and humanities courses:

1. **Include People with Lived Experience:** Whenever possible, invite people with lived experience or their relatives to participate, and include films created by people with lived experience.
2. **Promote Transdisciplinary and Interprofessional Learning:** Invite physicians and health professionals, along with other professionals from non–medical fields.
3. **Ensure Balanced Representation:** When selecting films and inviting guests, prioritize balanced gender representation and include underrepresented and vulnerable perspectives.
4. **Highlight Global Perspectives:** Even in universities in the Global North, perspectives from the Global South and other regions should be represented through appropriate films and, where possible, guest speakers to foster a deeper understanding of global health.
5. **Engage a Diverse Student Body:** A diverse student body should actively shape program selection, broadening the range of films and ensuring their relevance for the wider student community.
6. **Discuss Representation’s Role:** Students on the organizing committee should discuss the role of representation in a cinemeducation course and its influence on the program.
7. **Utilize the Prism Model and Cinemeducation Framework:** The prism model and cinemeducation framework can guide the curation of film and guest selection, offer a theoretical framework, and assess the suitability of cinemeducation courses.
8. **Attend Film Festivals:** Students and curriculum developers of cinemeducation courses should attend film festivals to curate diverse and representative films.
9. **Network for Collaboration:** Cinemeducation instructors should network with national and international peers at relevant conferences or film festivals and participate in regular exchanges.

At our university, we discussed these implications with the current organizing committee members, and they included these as criteria for programming future cinemeducation events. Furthermore, we initiated an exchange at film festivals and started a network between cinemeducation projects from different universities.

#### Strengths and limitations

A key strength of our study is the inclusion of all previous M23C events in the retrospective evaluation, offering a comprehensive view of the curriculum’s development over time.

One potential limitation of our study is confirmation bias, as most authors were part of the M23C. However, this participatory research design provided an insider’s perspective, likely contributing to a more nuanced and informed interpretation of the data.

Another limitation is our reliance on historical social media data and the M23C website for data collection. This introduces the possibility of transmission errors and data loss over time, which could lead to availability or recall bias. For example, one M23C event’s guest list could not be reconstructed. To ensure accuracy, we thoroughly checked the data collection process multiple times.

As the study was retrospective, gender data for guests and producers were inferred from first names, which may not accurately reflect their true gender identities and potentially underrepresent non–binary individuals. We acknowledge this limitation and note that our gender classification may not fully capture the diversity of gender identities.

A limitation of our study is the inability to determine exact viewer numbers for all M23C events. While social media and website data suggested varying attendance, precise figures could not be reconstructed, limiting our evaluation of student reception. We relied solely on available data, but accurate statistics remain unavailable.

Our study examines only a subset of cultural competence and touches on intersectionality only in passing due to limited data. Future research on cinemeducation should therefore not only explore the broader dimensions of cultural competence but also rigorously address intersectional dimensions and their impact on learners’ perspective taking.

This study is based on a limited understanding of diversity, mainly due to the retrospective nature of the data analysis. Moving forward, diversity should encompass more than gender, academic background, and geographic representation; it should be defined as disadvantaged groups within a population and consider intersectionality [55]. Prospective studies could, for instance, include parameters such as sexual orientation or gender identity, educational or socio–economic disadvantages, rural residency, migration history, single parenting, caregiving responsibilities, or being native.

### Conclusion

Cinemeducation has proven to be a versatile teaching method for addressing a wide range of medical humanities topics through reflective learning and perspective–taking. This study highlights the need for more inclusive programming that prioritizes perspectives from the Global South and underrepresented voices, including female and non–binary directors and people with lived experiences. To achieve this, future cinemeducation initiatives should actively engage diverse health and non–health students in organizing committees and integrate films from diverse cultural and geographic backgrounds. Additionally, attending international and regional film festivals could facilitate the discovery of representative and thought–provoking films. Future research should systematically compare cinemeducation programs globally to refine best practices and strengthen their role in medical education.

## Supplementary Material

Supplementary Material Supplementary_File_7_Detailed_overview_of_all_M23C_events.xlsx

Supplementary Material Supplementary File 1 Translations.xlsx

Supplementary Material Supplementary File 2 Topics of the M23C.xlsx

Supplementary Material Supplementary File 3 Geographic distribution.docx

Supplementary Material Supplementary File 6 Guests institution by gender.xlsx

Supplementary Material Supplementary File 4 Production countries.xlsx

Supplementary Material Supplementary File 5 Guest characteristics.xlsx

## Data Availability

This publication and its supplementary files include all data generated or analyzed during this study–except the names and associated titles of the guests, which we have not included for data protection reasons.
