# Supplementary material for: Cinemeducation: a descriptive mixed-methods analysis of perspectives in a medical humanities course
Source: Med Educ Online. 2025 Nov 18;30(1):2579077. doi: 10.1080/10872981.2025.2579077 (PMC12632234; doi:10.1080/10872981.2025.2579077)
Supplement: Supplementary Material — Supplementary File 3 Geographic distribution.docx [file ZMEO_A_2579077_SM3761.docx]

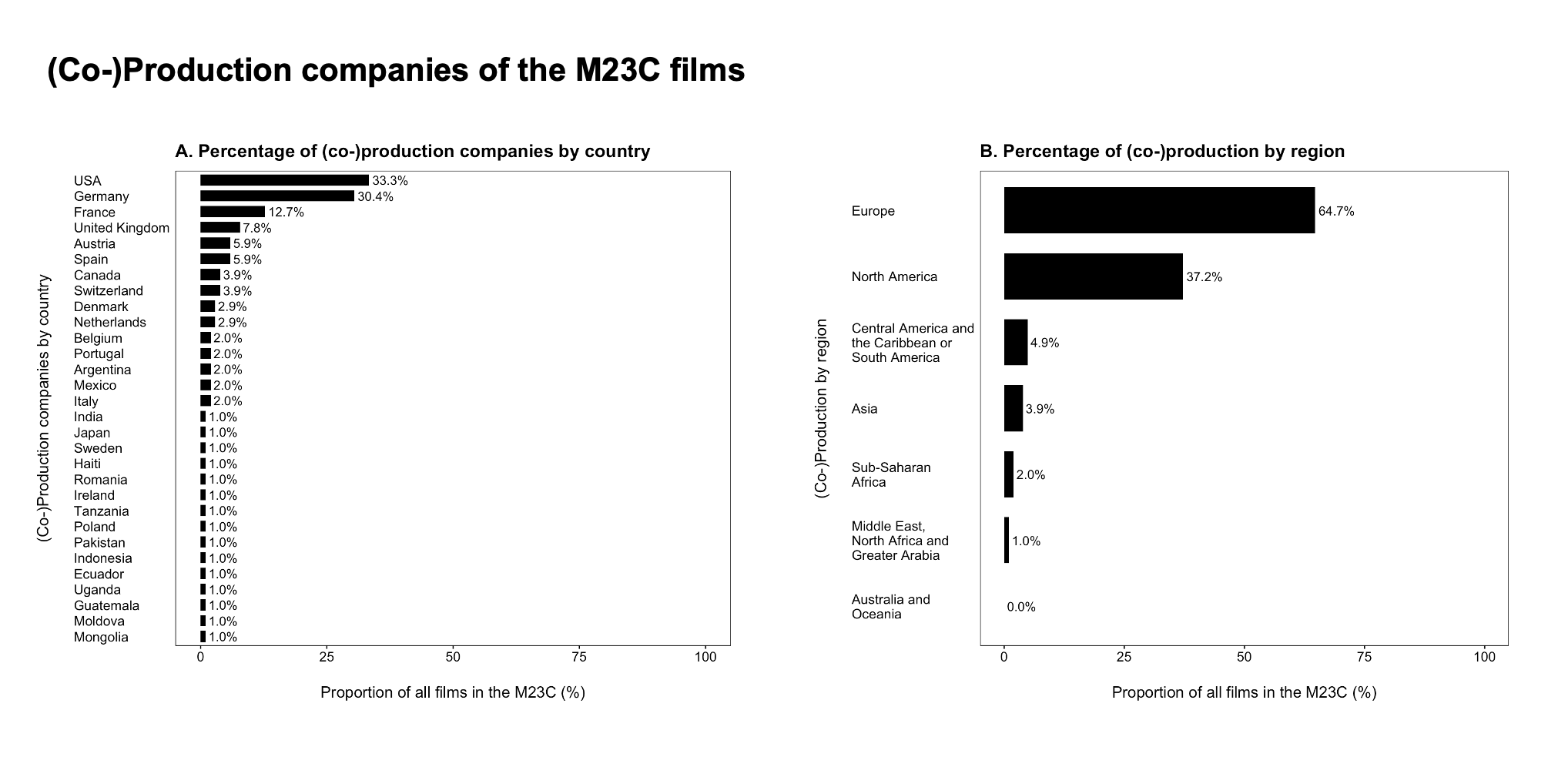


**Figure S1: Geographic distribution of (co-)production companies for films shown at the M23C**

Panel A shows the percentage of (co-)production companies by country for films included in the M23C evenings, highlighting the prominent role of the USA (33.3%) and Germany (30.4%) in the (co-)production of these films. Panel B groups (co-)production by region, showing Europe (64.7%) and North America (37.2%) as the primary contributors.
